# Gun-shot Wound through Left Tarsus—Amputation in Lower Third of Leg, More Than Six Months after the Receipt of the Injury—Case Occurring in Stanton U. S. A. General Hospital, Surgeon John A. Lidell, U. S. V. in Charge

**Published:** 1864-04

**Authors:** Wm. H. Gail


ART. II.—*Gun-shot wound through left Tarsus—Amputation in lower third of leg, more than six months after the receipt of the injury—Case occurring in Stanton U. S. A. General Hospital, Surgeon* JOHN A. LIDELL, *U. S. V. in charge—Reported by* WM. H. GAIL, M. D.

Private, Frederick Stackpole, Co. A, Sixth Maine Volunteers, aged 20 years, and of sound constitution, was wounded in the left foot by a conical leaden bullet in the battle of Fredericksburg, Va., May 3d, 1863. The missile passed through the tarsus in an oblique direction from within, outwards, and from before backwards. It entered in the neighborhood of the internal cuneiform bone, and escaped a short distance below the external malleolus, fracturing the bones of the tarsus in its passage. The ankle joint was not involved. He was admitted to Stanton Hospital May 6th, 1863. The injured foot was then inflamed, hot, and swollen to about twice its natural size; but the patient's general condition was good. It was deemed advisable to attempt to save the limb, and with a view to accomplish that result, the ice dressing was prescribed for the foot, together with a good diet and quietude.

May 8th. Suppuration commenced; ordered nutritious diet and milk punch.

May 10th. Slight bleeding from wound, which was stopped by the Liq. Ferri, persulph. on lint.

May 20th. Pus collected in the sole of the foot; liberated it by several incisions through the plantar fascia.

During the month of June no good change occurred. The discharge from the wounds and incisions continued healthy; a few pieces of bone, small in size, also came away. He was cheerful, and had a good appetite; pulse natural; the tumefaction of the foot gradually assumed an indurated character.

During the month of July there were occasional inflammatory exacerbations of the foot. The excessive heat of the weather also debilitated him somewhat. ℞ Tinct. Ferri, Mur. gtt. xv, three times a day.

In the course of the month of August several pieces of carious bone, varying in size from a pea to a hickory nut were removed at different times, The discharge remained about the same in quantity and of good quality. Tried compression of the swollen foot by means of a bandage, but derived no advantage from it. Patient's spirits and appetite good. On pleasant days he leaves the ward to get the benefit of out-door atmosphere.

About the middle of September he had a diarrhœa for three days, which was controlled with bismuth sub. nit.

In the course of the month of October and the fore part of November he recovered entirely from the debility engendered by the excessively hot weather of the summer and the diarrhœa of September, but the foot did not improve; it continued swollen to about twice the natural size, and was very hard in feeling. The orifices of the gun-shot wound remained open, and a probe could readily be passed from the one to the other through the tarsus; the probe also detected a good deal of bare bone in the same locality. The discharge had become thin, serous and straw colored. The patient had become rather thin in flesh, but in every other respect his condition was good.

There being no prospect of making the foot useful, it was amputated, by the circular method, in the lower third of the leg, by Surgeon John A. Lidell, November 24, 1863, the patient being under sulphuric ether. But little blood was lost, and but little "shock" exhibited; some hemorrhage occurred from the stump after it had been dressed. It was opened, a small amount of coagulum removed, and the bleeding stopped by Liq. Ferri, per sulph. applied on scraped lint. The principal source of the hemorrhage was a small artery in the medullary canal of the tibia, and another in that of the fibula.

Dec. 4th. Patient in good spirits and progressing well in every respect.

